# Histological Remission of Eosinophilic Esophagitis Following Allogeneic Hematopoietic Stem Cell Transplantation for Acute Myelogenous Leukemia

**DOI:** 10.7759/cureus.60687

**Published:** 2024-05-20

**Authors:** Gabriela Méndez, Theodor Griggs, Daniel Ludi, Cemal Yazici, Su Yeon Yeon

**Affiliations:** 1 Internal Medicine, University of Illinois at Chicago, Chicago, USA; 2 Gastroenterology and Hepatology, University of Illinois at Chicago, Chicago, USA; 3 Pathology, University of Illinois at Chicago, Chicago, USA

**Keywords:** endoscopy, histological remission, acute myelogenous leukemia, hematopoietic stem cell transplantation, eosinophilic esophagitis

## Abstract

Eosinophilic esophagitis (EoE) is an immune/antigen-mediated disease with an increasing incidence over the last decade. Clinicopathological remission can be achieved through different treatment options but often requires chronic therapy. To our knowledge, this is the first report of EoE wherein the patient (a 54-year-old man) achieved histological remission after allogeneic hematopoietic stem cell transplantation (HSCT) for acute myelogenous leukemia. Overall, despite the success of EoE treatment in this case, further studies are needed to establish allogeneic HSCT as a curative option for EoE.

## Introduction

Eosinophilic esophagitis (EoE) is a chronic disease characterized by immune/antigen-mediated processes. Several lines of evidence support the theory that EoE is mediated by type 2 helper T cell activity and is primarily induced by food allergens, leading to esophageal eosinophilia and remodeling of the esophageal epithelium [1]. Although various treatment options are available, including proton pump inhibitors (PPIs), swallowed topical corticosteroids, dupilumab, a 6-food elimination diet, and elemental diets, it is crucial to maintain consistent therapy to prevent relapses [2,3]. In addition, elimination diets are restrictive and difficult to follow.

Over the last two decades, allogeneic hematopoietic stem cell transplantation (HSCT) has gained wider acceptance for the management of difficult-to-treat autoimmune diseases [4]. We present the case of a 54-year-old man with EoE who achieved histological remission after undergoing allogeneic HSCT for acute myelogenous leukemia (AML). To our knowledge, successful EoE treatment using allogeneic HSCT has not been reported previously, although the converse has been reported in pediatric populations [5].

## Case presentation

**Figure 1:**
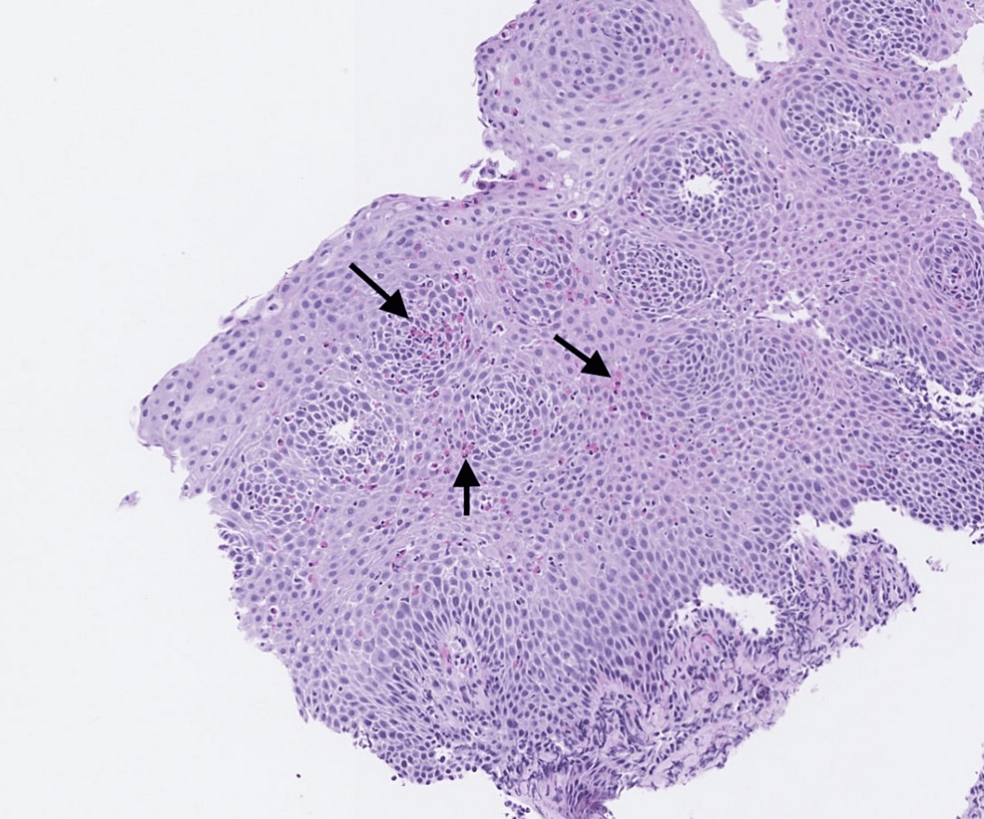
Histopathology image Intraepithelial eosinophils (black arrows) infiltrating mostly the superficial squamous epithelium of the mid-esophagus.

A 54-year-old man with Graves’ disease and hidradenitis suppurativa, experiencing intermittent episodes of dysphagia in the mid-chest region as well as heartburn, visited our clinic in 2016. With no prior endoscopy, an upper endoscopy was pursued for further evaluation. Endoscopic findings were significant for esophageal mucosal changes with longitudinal furrows in the middle and lower thirds of the esophagus, suggestive of EoE. Mid-esophageal biopsies further confirmed the diagnosis of EoE, with numerous intraepithelial eosinophils with a cell count of 25-43 eosinophils per high-power field (HPF) exhibiting degranulation and clustering (Figure 1). The patient declined treatment with PPIs as he preferred symptom monitoring. Upon re-evaluation after six months, he reported experiencing 1-2 episodes of dysphagia per month. He stated that he ate slowly and used liquids to ingest solids properly. The intended treatment plan involved a six-food elimination diet and 40 mg of PPIs daily, both of which are first-line treatment options. However, after receiving a diagnosis of AML in September 2017, the patient was lost to follow-up.

After AML diagnosis, an induction regimen was initiated with cytarabine and daunorubicin. A bone marrow biopsy (BMB) conducted 14 days after treatment initiation revealed recurrent blast cells. Chemotherapy with mitoxantrone, etoposide, and cytarabine was initiated; however, CD34 immunohistochemistry revealed areas with up to 10% blast cells. The patient was discharged with subsequent treatment plans involving matched, unrelated donor peripheral blood stem cell transplantation (SCT). Upon readmission, the patient underwent pre-transplantation conditioning with palifermin, fludarabine, basulfan, and TMI. Additionally, tacrolimus and methotrexate were administered for graft-versus-host disease (GVHD) prophylaxis. After allogeneic HSCT, the patient was discharged under oral tacrolimus treatment (2 mg, twice daily). Six months after the transplantation, BMB revealed AML remission. Subsequently, the tacrolimus dose was tapered with no signs or symptoms of GVHD.

**Figure 2:**
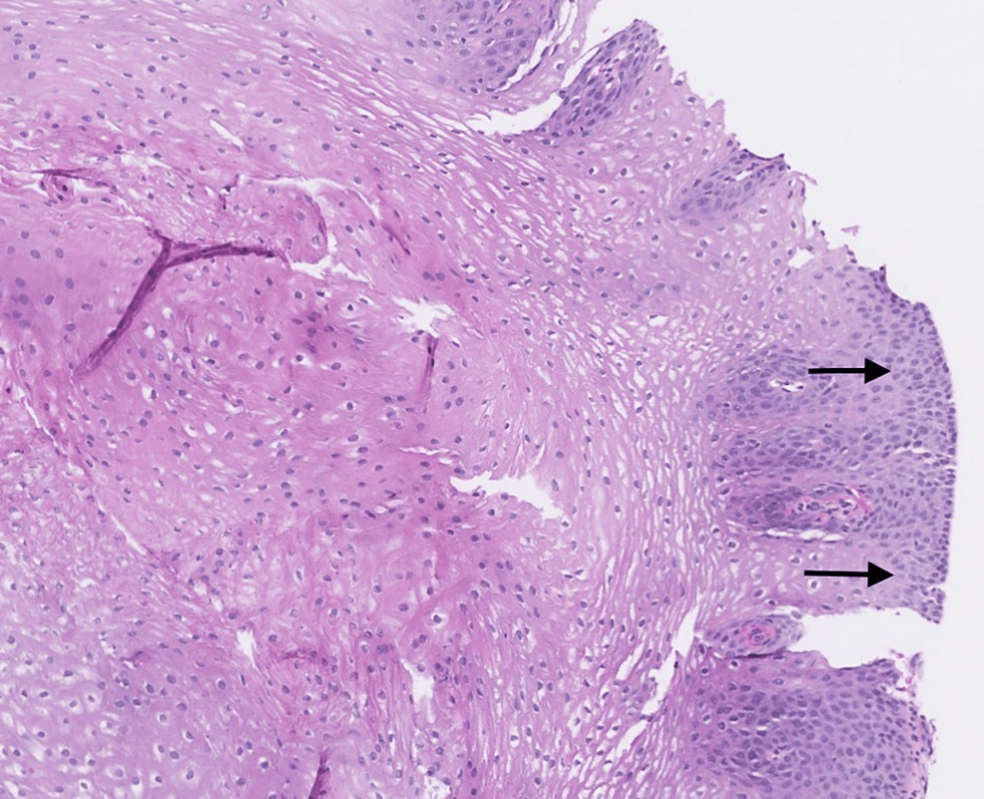
Biopsy of the proximal esophagus The image shows squamous epithelium (black arrows) without histopathological changes, including eosinophilic infiltrate; Barret mucosa, intestinal metaplasia, dysplasia, and neoplasia.

**Figure 3:**
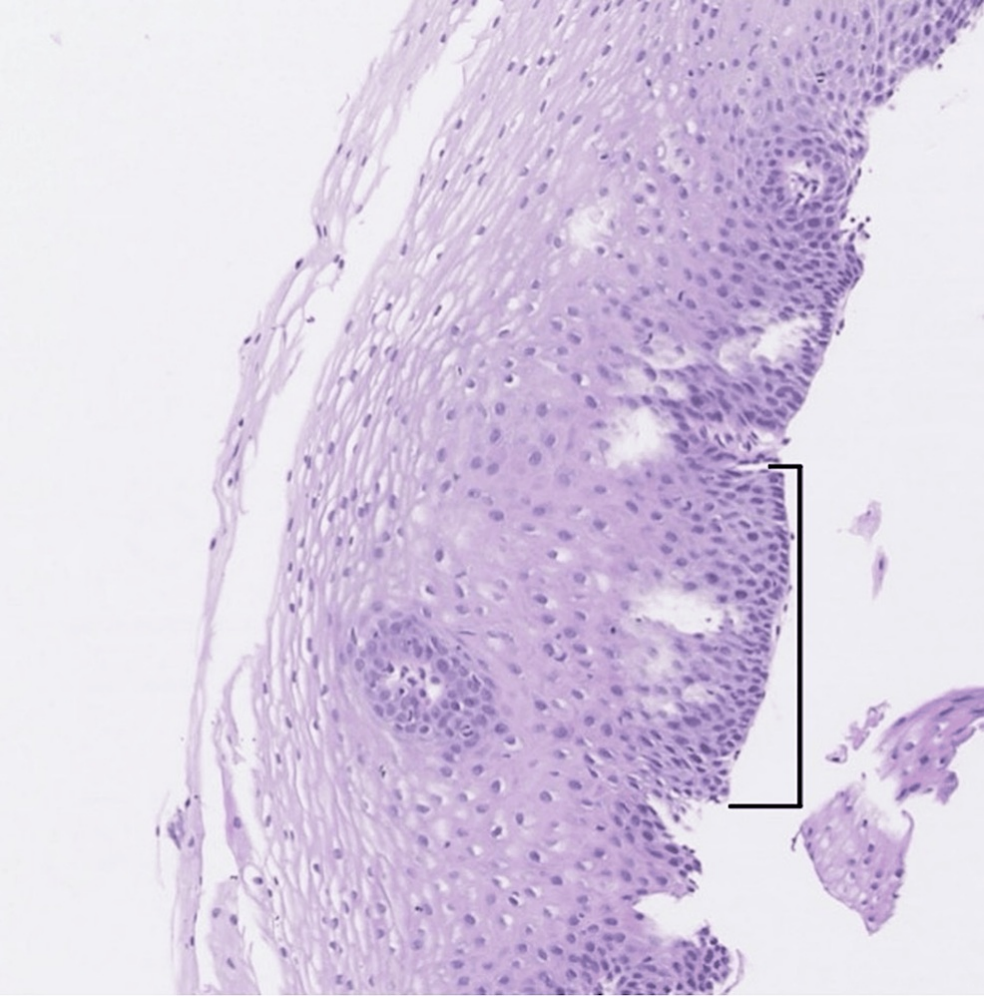
Biopsy of the distal esophagus The image shows squamous mucosa with mild papillomatosis (black bracket) compatible with reflux esophagitis but negative for eosinophilic infiltrate, Barret mucosa, intestinal metaplasia, dysplasia, and neoplasia.

The patient returned to the clinic in 2023 for colorectal cancer screening. He reported symptoms of dysphagia, experiencing a sensation of food blockage in his mid-chest every few months. The patient denied current use of PPIs but reported previous usage for acid reflux and not EoE, suggesting nonadherence with the previously recommended regimen. Upper endoscopy performed in 2023 did not reveal any evidence of esophagitis, stenosis, or linear furrows in the esophagus. Biopsies of the proximal (Figure 2) and distal (Figure 3) esophagus revealed a squamous mucosa with changes similar to those observed in reflux esophagitis and, significantly, did not show elevated levels of eosinophils. Gastric biopsies revealed active chronic gastritis with *Helicobacter pylori* infection. The patient was treated with triple therapy for the *H. pylori* infection with levofloxacin 500 mg, amoxicillin 500 mg and omeprazole 20 mg with plans to follow up to confirm *H. pylori *eradication.

## Discussion

Eosinophilic esophagitis is a Th2 antigen-driven disease in which chronic eosinophil-rich inflammation causes esophageal dysfunction. The clinical presentation of EoE varies depending on age at presentation. As in our patient, adolescents and adults typically have symptoms associated with esophageal fibrosis, with more than 70% of adults presenting with dysphagia and 30% presenting with food impactions [6]. Currently, 15 eosinophils or more per HPF in the maximally affected high-power field is required for diagnosis [6].

In EoE, the goal of therapy is to improve symptoms and reduce the eosinophil count below 15 eosinophils per HPF [6]. Current treatment options include PPIs, swallowed topical corticosteroids, dupilumab, an elemental diet, and a 6-food elimination diet. The effectiveness of these treatment options in achieving a histological response has been shown to be 41.7%, 64.9%, 85%, 93.6% and 67.9%, respectively [6,7]. Furthermore, it is critical to remember that EoE is a chronic disease that requires long-term therapy in most patients in order to prevent symptomatic, endoscopic, and histologic relapse [2]. Therefore, it would be less likely that the previous use of PPIs in our patient was the definitive cause of histological remission, given he was not taking PPI therapy at the time of reevaluation. 

Although not a first-line treatment option, HSCT has been shown to be effective in various gastrointestinal diseases, including Crohn’s disease (CD), refractory celiac disease, and chronic liver diseases [4]. However, there have been no reported instances of achieving histological remission in EoE following HSCT, making our case unique. In allogeneic HSCT, the conditioning regimen eradicates malignant, ineffective, hematopoietic, and host immune cells [8]. Subsequently, stem cell transplantation provides a new and effective immune system with regeneration of naïve T-lymphocyte derived. Taking these findings together, we hypothesize that ablation of our patient's immune system and reconstitution with the donor stem cells resulted in eosinophilic histological remission. Nonetheless, the immunosuppressive treatment used to prevent GVHD may have also influenced the clinical course of histological remission in our patient. However, tacrolimus was discontinued five years prior to the endoscopy which demonstrated histological remission. Thus, the likelihood of histological relapse increases due to the absence of immunosuppression.

Interestingly, despite achieving histological remission, our patient still endorsed symptoms of dysphagia and food impaction. While this could be secondary to acid reflux, it may also suggest that while allogeneic HSCT could be a treatment option for histological remission, it may not be effective for symptomatic resolution. Moreover, it is important to note that chronic inflammation in the setting of active EoE can lead to fibrosis and chronic intermittent dysphagia despite the resolution of active disease which may underly the patient’s current presentation. Further prospective studies are necessary to determine whether allogeneic HSCT with a conditioning regimen can successfully achieve clinico-histological remission in patients with EoE. The mechanism is that the host immune system is destroyed by conditioning regimens, and a new immune system is replaced by allogeneic HSCT. Thus, this treatment prevents the recurrence of esophageal eosinophilia while simultaneously alleviating the burden associated with medication compliance.

## Conclusions

Eosinophilic esophagitis is an immune/antigen-mediated disease with increasing incidence over the last decade. It is characterized clinically by esophageal dysfunction manifesting as dysphagia and food impaction. Standard therapy includes PPIs, swallowed topical corticosteroids, and elimination diets. While all these treatment options have shown to be effective in achieving clinico-histological remission, discontinuation can lead to symptomatic and/or histological relapse. In the case presented, histological remission of EoE following allogeneic HSCT was achieved. Further prospective studies are necessary to determine if allogeneic HSCT using a conditioning regimen can become a treatment option to achieve clinico-histological remission in patients with EoE.
